# Social and seasonal variation in dwarf mongoose home-range size, daily movements, and burrow use

**DOI:** 10.1093/beheco/arae082

**Published:** 2024-10-05

**Authors:** Josh J Arbon, Amy Morris-Drake, Julie M Kern, Luca Giuggioli, Andrew N Radford

**Affiliations:** School of Biological Sciences, University of Bristol, Tyndall Avenue, Bristol, BS8 1TQ, United Kingdom; School of Biological Sciences, University of Bristol, Tyndall Avenue, Bristol, BS8 1TQ, United Kingdom; School of Environmental and Rural Science, University of New England, Elm Avenue, Armidale NSW 2351, Australia; School of Engineering Mathematics and Technology, University of Bristol, Tyndall Avenue, Bristol, BS8 1TQ, United Kingdom; School of Biological Sciences, University of Bristol, Tyndall Avenue, Bristol, BS8 1TQ, United Kingdom

**Keywords:** costs of reproduction, group size, home range, movement, NDVI, seasonality

## Abstract

When making decisions about resource use, social species must integrate not only environmental factors but also the influence of opportunities and costs associated with group living. Bigger groups are expected to move further and to need access to larger areas for adequate food acquisition, but the relationships with group size can vary seasonally and with reproductive stage. Shelters are often more consistent in availability than food, but their use relates to factors such as predator defense and parasite transmission that are themselves influenced by group size and seasonality. Here, we used long-term data to investigate resource use and associated movement in a wild population of dwarf mongooses (*Helogale parvula*). We found that bigger groups occupied larger home ranges, moved larger daily distances and covered more daily area than smaller ones, while environmental greenness (measured by normalized difference vegetation index [NDVI]) influenced daily movements in the breeding season but not the non-breeding season. Both assessed axes of seasonality also had pronounced effects on shelter use: mongoose groups used more unique sleeping burrows, and switched between burrows more often, in the breeding season, but also switched more when environmental greenness was higher. By investigating specific periods within the breeding season, we revealed the constraints that vulnerable, poorly mobile offspring impose on both group movements and burrow use, highlighting a potentially overlooked cost of reproduction. Our results show how both social and environmental factors can affect key resource-use decisions, demonstrating potential costs and benefits to group living within distinctly seasonal geographic areas.

## Teaser

Group size, seasonality, and reproductive stage influence the use of key food and shelter resources in dwarf mongooses. Group size appears most important when considering foraging, as bigger groups occupy larger areas and move further than smaller groups to satisfy energetic needs, but all groups are constrained by dependent pups. Seasonality has more pronounced effects on burrow use as groups use more sleeping burrows and switch between burrows most often during the breeding season and when the landscape is greener.

## Introduction

Animals must make decisions about resource use, such as where to find food and shelter. For solitary species, those decisions are predominantly driven by environmental factors; for example, there is a well-established link between resource availability and home-range size in a diversity of taxa (Marshall and Cooper 2004; Stehle et al. 2017; Rodríguez‐Recio et al. 2022), while seasonality can affect the use of shelters (Hyslop et al. 2009). For group-living species, social factors are also likely to play an important role. Group members can facilitate the use of resources, such as through cooperative foraging (Bednarz 1988; Boesch 1994), predator repulsion (e.g. by mobbing Graw and Manser 2007; Kern and Radford 2016), and defense against conspecific outsiders (Radford 2003; Willems et al. 2015). However, sociality also entails costs as group members often compete for the same resources (Janson 1992; Grand and Dill 1999; Krause and Ruxton 2002; Jones et al. 2009). As such, the home ranges inhabited by animal groups—often referred to as a territory when exclusively defended (Börger et al. 2008)—and resource use within those areas are liable to be affected by both social and ecological factors. Investigating these relationships will further our understanding of how social animals access key resources in their environment whilst trading off the costs and benefits of group-living.

One primary resource that animals require is food. It has long been suggested that bigger groups need to explore larger areas because they deplete foraging patches faster (McNab 1963; Altmann 1974). This general principle is captured in the ecological constraints model of group size (Chapman and Chapman 2000a), as larger groups need to travel further to satisfy their resource needs, and the costs of such travel ultimately constrain group size (Chapman and Chapman 2000b). Habitat quality can therefore influence group movements through resource availability and it logically follows that, habitat quality being equal, bigger groups should occupy larger home ranges due to their increased energetic demands (Boinski and Garber 2000). Recent work has highlighted how other pressures on individuals within groups can modify the relationship between group size and movements. For instance, there is a quadratic relationship between group size and home-range size for vulturine guineafowl (*Acryllium vulturinum*), where intermediate groups have the largest home ranges (Papageorgiou and Farine 2020). This may arise because the movements of large groups present a collective action problem whereby the different interests of individuals cause divergence from optimal behavior (Strandburg-Peshkin et al. 2015; Papageorgiou and Farine 2020). An opposite nonlinear relationship was observed in baboons (*Papio cynocephalus*), where groups of intermediate size have the smallest home ranges and daily travel distances as small groups moved further to avoid predators and larger rivals (Markham et al. 2015).

The level of competition can also be affected by resource availability (Hubbs and Boonstra 1998; Corriale et al. 2013): an increase in food (such as following rains: Denlinger 1980; D’Souza et al. 2021) will result in a group needing to move less far to satisfy its energetic needs (Kittle et al. 2015). As seasonality can affect resource availability, animals may change their behavior to buffer against changes between seasons (Lindström 1989; Papageorgiou et al. 2021). For example, normalized difference vegetation index (NDVI)—a remote-sensed measure of vegetation productivity (e.g. greenness)—was negatively related to home-range size across carnivores (Duncan et al. 2015) and ungulates (Seigle-Ferrand et al. 2021), such that species used smaller home ranges when the landscape was greener and therefore more productive. Iberian ibex (*Capra pyrenaica*) used smaller home ranges in seasons when resource availability was more stable (Viana et al. 2018), while meerkat (*Suricata suricatta*) territories were able to accommodate larger groups in more resource-abundant years (Bateman et al. 2015).

Shelter represents another key resource for many species (Sheets et al. 1971; Kowalczyk et al. 2004; Naďo and Kaňuch 2015; Bose et al. 2020). Many animals use multiple shelters within their home range including those for sleeping, breeding, and predator avoidance (Manser and Bell 2004: 200; Mautz et al. 2011; Strandburg-Peshkin et al. 2019). Although shelter availability fluctuates less than food sources during the year, the costs and benefits associated with their use are also likely to be affected by ecological and social factors such as seasonality and group size. Seasonality can be associated with changes in parasite abundance, such as an increase following rainy periods (Rechav 1982; Archer et al. 2014; Lutermann et al. 2015). Shelters can act as reservoirs of ectoparasites, as well as hubs of pathogen transmission, and therefore may be avoided or switched when pathogenic and parasitic pressures are high. Such behavioral mitigation has been demonstrated in multiple species: for instance, Brant’s whistling rats (*Parotomys brantsii*) were much less likely to switch burrows when their parasite load was experimentally reduced (Roper et al. 2002). Parasite avoidance has also been demonstrated to aid fitness: African ground squirrels (*Xerus unauris*) had their breeding output greatly increased by the experimental removal of parasites despite no obvious effects of parasite load on adult health (Hillegass et al. 2010). Moreover, switching between sleeping locations has been demonstrated as an effective anti-predator strategy in multiple primate (Smith et al. 2007; Ramanankirahina et al. 2012) and non-primate (Strandburg-Peshkin et al. 2019) species. Repeat use of a sleeping site can lead to the buildup of cues of group presence, such as scent, and allow predators to recognize predictable patterns in behavior that may lead to increases in predation risk. In relation to both parasite and predation risk, the need for shelter switching might be greater for larger groups. This is because a large number of individuals increases the chance of parasite acquisition (Rifkin et al. 2012; Patterson and Ruckstuhl 2013) and might be more detectable by predators (Dehn 1990; Riipi et al. 2001).

In many species, seasonal changes are also associated with major life-history events such as breeding (Hockey et al. 2005). These events alter the social composition of groups, having knock-on effects for their ability to move quickly and cohesively. Vulturine guineafowl groups with more chicks have been observed to occupy smaller home ranges, suggesting groups were constrained to stay within a certain area (Papageorgiou and Farine 2020); however, daily movement distances were not impacted by the presence of chicks. In contrast to precocial young, many mammal species produce altricial young that remain sheltered for their early life. This offspring vulnerability can limit adult behavior as shown by female badgers (*Meles meles*) that used fewer setts whilst breeding with their dependent offspring remaining in one location (Rosalino et al. 2005), restricting access to alternative resources. Despite the many studies into the movements of species that produce altricial young, the potential hidden costs of reproduction on movement and resource use remain understudied.

Here, we use 10 yr of data from a habituated, wild population to investigate the effects of group size, breeding season, and environmental productivity on resource use and associated movement in dwarf mongooses (*Helogale parvula*). We used NDVI to partition the effect of changes in environmental greenness (and thus likely productivity) from that driven by breeding. Dwarf mongooses are cooperatively breeding mammals that live in family groups (Rood 1983), with each group defending a territory year-round (Christensen et al. 2016; Morris-Drake et al. 2019). Dwarf mongooses predominantly eat arthropod prey that they dig from the ground (Rasa 1986a) but will also opportunistically prey on lizards, snakes, birds, and small rodents. As such, food resources are widely distributed throughout the environment but are seasonally variable in their abundance (D’Souza et al. 2021). Dwarf mongooses breed in the warm, wet Austral summer (September to March) (Morris-Drake et al. 2023); during the rest of the year, it is cooler and drier and there is lower insect abundance (Finch and Meadows 2019; D’Souza et al. 2021). Dwarf mongooses are strictly diurnal, spending nights predominantly underground in excavated termite mounds (Rasa 1987), but also logs, hollow trees, and rock-piles (Hoffmann et al. 2014); the availability of these sleeping burrows is consistent across time. Mongooses are host to a suite of ectoparasites, including ixodid tick species that are associated with burrows (Horak et al. 1999) and are vulnerable to a range of terrestrial and aerial predators (Rasa 1989).

We predicted that bigger groups would have larger home ranges than smaller groups, and would move further and cover more area on a daily basis, to satisfy their greater energetic needs (McNab 1963; Altmann 1974). We also predicted that home-range size would be smaller, and that groups would need to travel less distance and cover less area per day, when the environment was greener and therefore contained more prey. We expected movement to be constrained by dependent offspring, resulting in reduced daily movements during the breeding season compared to the non-breeding season. We predicted that mongooses should switch their burrow more regularly and use more distinct burrows when the landscape is greener—switching burrows represents only a small portion of daily movement—to mitigate associated increases in parasites, with this effect predicted to be especially strong in the breeding season to protect vulnerable offspring (Butler and Roper 1996; Hockey et al. 2005; Lutermann et al. 2015). We also predicted that, compared to smaller groups, bigger groups should use more burrows and switch more often due to their increased detectability to predators and parasite transmission potential (Dehn 1990; Riipi et al. 2001; Rifkin et al. 2012; Patterson and Ruckstuhl 2013). Finally, we predicted that due to the high vulnerability of young offspring, groups with pups should switch burrows more frequently than those without to minimize detection, infection, and associated mortality.

## Methods

### Study species

Dwarf mongooses are cooperatively breeding mammals that live in groups of 3 to over 20 adults (individuals over 6 mo old that forage independently) plus dependent offspring. Groups are composed of a dominant breeding pair and both natal and immigrant helpers of both sexes (Kern et al. 2023). Each group defends a collective territory from rivals through scent-marking at latrine sites, as well as directly repelling intrusions (Christensen et al. 2016; Morris-Drake et al. 2019, 2021, 2023). Mongoose groups have multiple litters per breeding season—breeding seasons are defined as starting at the onset of estrus in the population and running until the last litter in the population has emerged from the breeding burrow for the first time (as in Morris-Drake et al. 2023); the remainder of the year is classified as the non-breeding season. Litters of pups are almost exclusively produced by the dominant pair, and any pups born to other mothers come from within the group and are born synchronously with those of the dominant female (Rood 1990; Arbon et al. 2024). This breeding dynamic results in up to 3 cohorts of differently aged pups each separated by approximately 2 mo. Mongoose pups remain at the burrow and are looked after by one or a few adults, known as babysitters, for the first month of their life (Rasa 1977; Rood 1978), while the remaining group members leave the burrow area to forage. Multiple adults within the group act as babysitters, and individuals often rotate within a day. At roughly 1 mo old, the pups start moving with the group, first being fed by adults and then beginning to forage independently.

Dwarf mongooses spend the majority of the daylight hours foraging, moving through the landscape as a cohesive group (Kern and Radford 2021). Mongoose groups spend each night in one of a series of burrows within their territory. At our study site, in the southern part of their range (North-Eastern South Africa), burrows are an abundant resource; many groups have been observed using over 100 unique sleeping locations over the 10 yr of observation (unpublished data). Dwarf mongooses are prey to many avian (African hawk-eagle *Aquila spilogaster*, martial eagle *Polemaetus bellicosus*, Wahlberg’s eagle *Hieraaetus wahlbergi*, tawny eagle *Aquila rapax*, steppe eagle *Aquila rapax*), mammalian (black-backed jackal *Aquila rapax*, caracal *Caracal caracal*, serval *Leptailurus serval*, white-tailed mongoose *Ichneumia albicauda*, banded mongoose *Mungus mungo*) and reptilian (rock monitor *Varanus albigularis*, rock python *Python natalensis*) predators.

### General data methods

All data were recorded at the Dwarf Mongoose Research Project (DMRP), Sorabi Rock Lodge, Limpopo Province South Africa (24.11 S, 30.46 E). Data were collected between September 2013 and September 2023, from 12 groups of wild dwarf mongooses habituated to the close presence of observers (<5 m proximity on foot). Groups were usually followed for 2 to 3 consecutive days per visit; each group was visited for a mean ± SE of 9.2 ± 0.1 d per month, with a gap of 5.8 ± 0.1 d between visits. Work was conducted under permission from the Limpopo Department of Economic Development, Environment and Tourism (permit number: 001-CPM403-00013), and ethical approval from the University of Pretoria, South Africa (Animal Ethics Committee: NAS321/2022) and the University of Bristol, UK (Animal Welfare and Ethics Review Body: UIN/17/074), and in line with ASAB guidelines for the ethical treatment of animals (ASAB 2020). Groups were followed by an observer from their morning sleeping burrow whilst they conducted their daily foraging, until they returned to their evening sleeping burrow. For September–May, observers would leave the group once they had ceased foraging activity in the morning, avoiding the daily peak temperatures, returning to find the group in the afternoon. The rest of the year, groups were followed continuously throughout the day. Group composition was recorded each day that a group was observed. Tracking data were obtained from Garmin Etrex 10 (Garmin, Olathe, USA) handheld GPS devices (resolution generally higher than 3 m), which were carried by the observers who placed themselves near the group centroid.

We carried out all data processing and analyses in R version 4.4.0 (R Core Team 2019). We calculated group sizes for daily variables (i.e. total track length, area covered, burrow switching) from the number of individuals noted as present in the group on the specific day. Group sizes for whole-season variables (i.e. home-range size and number of burrows used) were calculated as a weighted mean, whereby each day was given an inferred group size based on known data via linear interpolation. If a group was visited on day 1 and had an observed size of 12, then again on day 4 with an observed size of 10, days 2 and 3 (which were unobserved) were given an interpolated group size of 11. We then took the mean of these group sizes across each group season to generate the weighted metric. All group sizes were calculated for those individuals over 6 mo in age; at this age, individuals forage independently and can comfortably keep up with group movement, therefore rendering this cutoff a useful proxy for investigating movement and space-use processes.

We obtained normalized difference vegetation index (NDVI) data, a measure of landscape greenness and therefore productivity (Pettorelli et al. 2005), from the MODIS product “MOD13Q1” (Didan 2021), taken at 16-d, 250 m by 250 m resolution, using functions from the “MODISTools” R package (Hufkens 2023). NDVI values were reprojected to WGS 84 such that values ranged between −0.2 (minimum productivity) and 1 (maximum productivity) using functions in the R package “terra” (Hijmans et al. 2024). For analyses of both home-range size and burrow usage across a season (e.g. 2015 non-breeding season for group BW), we calculated the weighted NDVI mean across a home-range polygon for all NDVI values within the time window for which the home range was calculated. As the mean home range was 6.25× the size of an NDVI pixel, the relative contribution of each pixel was accounted for based on its NDVI value and the proportion of overlap between the home range and that pixel. For analyses of daily tracks, we took the mean of NDVI values for each fix within the track for the closest available date. Similarly, for analyses of burrow switching, we used the NDVI value of the evening sleeping burrow from the closest available date. Temporal variation in NDVI was much larger than spatial variation, highlighting the distinct vegetation seasonality at the study site: mean standard deviation in NDVI across time at a given location was 0.13, whereas it was 0.03 at a given point in time (Fig. S1). Although the highest NDVI values (highest greenness) occurred during the breeding season, the first month of the breeding season (September) is one of the least green, and the first month of the non-breeding season (April) is among the greenest. Combined with annual variation in amount and timing of NDVI, this enabled us to partition out the effect of environmental greenness (as a proxy of productivity) when investigating changes related to breeding season.

When splitting data within breeding seasons, we focused on the first litter per group per breeding season to prevent potential confounds of groups having pups of overlapping litter ages within a season (i.e. while the second litter is at the burrow, the first one is still dependent and moving with the group). We retained for analysis any first litters where we knew the birth date with accuracy of within 1 wk. For litters born on days without an observer present, we took the median date between the date that the litter was first observed and the last date that the group was seen with the dominant female pregnant. We used known or calculated birth dates to generate 3 distinct periods: the *Pre* window as the 28 d before the litter was born (median month = October); the *Burrow* window as the 28 d following their birth when the pups are constrained to stay at the burrow (median month = November); and the *Emerged* window as the following 28 d (29 to 56 d post-birth) when the pups begin to travel with the group (median month = December).

We subsampled GPS fixes to attain a mean inter-fix interval of one minute following the procedures outlined in Langley et al. (2024). Track data were cleaned by trimming tracks between the time that the mongoose group left a burrow in the morning and arrived at a burrow in the evening. We excluded any track from a data-collection session that contained a sampling gap of longer than 15 min; DMRP baseline data collection involves GPS-marked behavioral scans every 15 min. Clear outliers and aberrant fixes, such as those that were clearly outside the possible space (e.g. the fenced reserve) were removed.

### Home-range size, daily movements, and burrow use

We estimated home-range sizes using 95% kernel density estimates (KDEs), calculated with the R package adehabitatHR (Calenge and Fortmann-Roe 2023). A minimum of 20 sampling days per group per season were required for an estimate to be generated; this cutoff was chosen as a trade-off between retaining a large number of group-season estimates, but removing low-sampling-effort seasons that result in largely unrepresentative estimates. We also removed 2 group-season estimates that were clear outliers (double the next largest estimates) as the group was not displaying home-ranging behavior but instead was roaming widely across the study site. We still included number of sampling days in our modeling to account for any potential residual relationship between sampling effort and KDE estimation after the 20-d cutoff. We also separately ran the home-range models with home-range estimates derived from an autocorrelated kernel density estimator (AKDE) due to potential effects of nonindependence of GPS fixes. KDE and AKDE estimates were strongly correlated (see Supplementary Material: Kernel Density Estimation; Fig. S2) and these methods generated qualitatively identical results for all predictors of interest (Tables S1 and S2).

For calculation of daily movement parameters, we took all days for which GPS tracking was started at the morning burrow and ended at the evening burrow. We then filtered these tracks to include only those for which the last fix of the morning session and the first fix of the afternoon session were separated by a maximum of 50 m, representing inactivity of the group in the intervening time (*n*_GroupDays_ = 1,192). In the summer months, mongooses will often leave their burrow to forage, then lay inactive for the hot middle hours of the day, resuming activity later in the afternoon as the temperature drops. This 50 m cutoff distance was chosen as it represents the maximum spread over which a foraging group of mongooses is regularly extended. This process prevents exclusion of days where the group did not meaningfully move between observation periods, but instead the observer ended the morning session in a different location within the group to the start of the afternoon session. It is worth noting that this thresholding may filter out days where mongooses were more active and moved further than this distance between sessions, although the number of tracks removed was even across months. The final analysis sample of 1,192 d was spread across all groups and seasons: each group contributed a mean ± SE of 99.8 ± 21.4 tracks (range = 7 to 207) while each season accounted for 57.0 ± 7.4 tracks (range = 6 to 144).

**Fig. 1.**
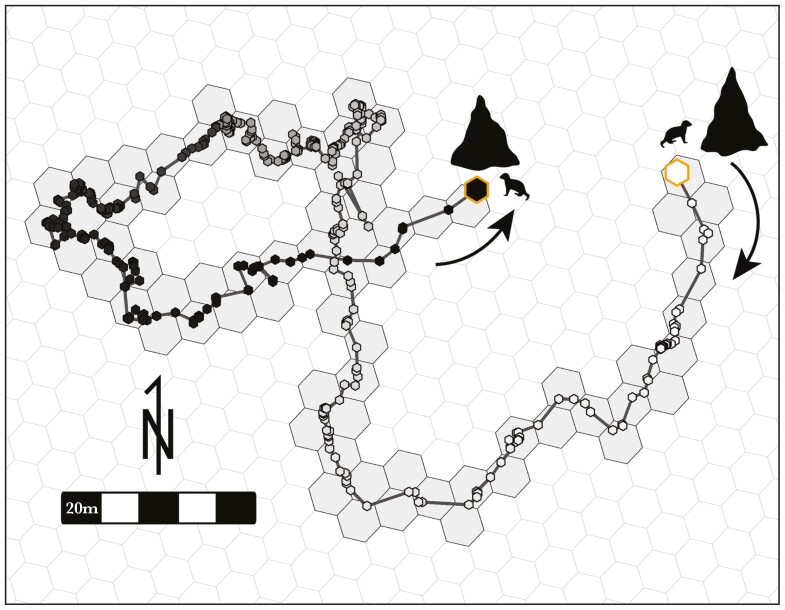
Graphical representation of metrics of a sample track. Gold-outlined points highlight the start (white) and end (black) of the day, with the color transition between the 2 representing progressing time. Total track length is measured as the sum of the distances between each fix (individual points), and area covered is calculated as the number of hexagons intersected by the track (black outline, gray fill).

We calculated total track length as the sum of distances between all fixes on a given day (Fig. 1), assuming that a straight line was traveled between fixes. To calculate area covered, we created a hexagonal raster of size 20 m, a distance representing the average spread of a dwarf mongoose group whilst foraging. For each track, the st_intersect() function from the “sf” R package (Pebesma 2018) was used to calculate the number of hexagonal cells that the track crossed (Fig. 1). As the area covered represents the space that the group moves across during the day, repeated use of an area does not result in an increase in the metric. This enables the capture of nuance in movement patterns that may not be represented by track length or other possible metrics such as maximum displacement distances. While a group could move back and forth between two areas, resulting in a large track length, it would not lead to a high total area covered. Conversely, groups could have a low displacement distance in any one direction but use all the territorial area inside that distance. Area covered thus allows us to differentiate whether a group used all the available space, or simply repeatedly used the same space, both of which could be true of a large total track length.

We defined the number of burrows used as the unique number of locations that a group used for overnight sleeping across a season. For days on which a group was observed at the morning and evening sleeping burrow, we noted whether the same or a different burrow was used. As with calculations of home-range size, we implemented a 20-d minimum cutoff to ensure representative samples of each season were being analyzed.

### Statistical methods

Using the package “lme4” (Bates et al. 2015), all models were fitted as linear mixed models with Gaussian error structures, with the exception of those investigating burrow switching, which were fitted as binomial generalized linear mixed models with a logit link function. We checked model diagnostics with the “DHARMa” package (Hartig 2022) using the testDispersion() and simulateResiduals() functions, as well as for variance inflation using the vif() function from the package “car” (Fox and Weisberg 2019). In all cases, model simplification to generate a final model entailed removal of nonsignificant interaction terms using an α of 0.05 to aid interpretation of main effects; main effects selected a priori were always retained in models. We calculated *P* values for fixed factors via likelihood ratio tests between the final model (after potential interaction term removal) and the final model plus/minus the term of interest. For pairwise comparisons between multilevel factors, we conducted post-hoc Tukey tests using the lsmeans() function from the “emmeans” package (Lenth 2024).

Full (global) models were fitted with season type (breeding vs. non-breeding), group size (weighted or daily depending on response variable), NDVI, and their interactions as fixed factors, as well as group identity and specific season (e.g. 2021 breeding season) as random factors (12 unique groups, 20 unique seasons across the dataset). Group size and NDVI metrics were scaled and centered. For models measuring whole season characteristics (home-range size and number of burrows used), number of days sampled was also added as a fixed term to account for sampling effort. To investigate the possibility of a group size optima as seen in other group-living species (Markham et al. 2015; Papageorgiou and Farine 2020), we tested for a quadratic effect of group size on home-range size and daily movement metrics by adding group size squared as a predictor to the final model. We also ran separate models for the same response variables (except the number of distinct burrows used, which lacked the necessary sample size per period) to investigate variation within the breeding season, including breeding-season period (*Pre, Burrow, Emerged*), group size and NDVI as fixed factors, and group identity and season as random factors. For all models investigating breeding-season period, a weak Bayesian prior was specified to aid with the fitting of the random effects structure of the model using the blmer()/bglmer() functions from the “blme” package (Chung et al. 2013). These act as wrapper functions for lme4 models, fitting a weakly informative prior to the covariance matrix (here a Wishart distribution), helping to avoid singular model fit.

## Results

### Home-range size

**Fig. 2.**
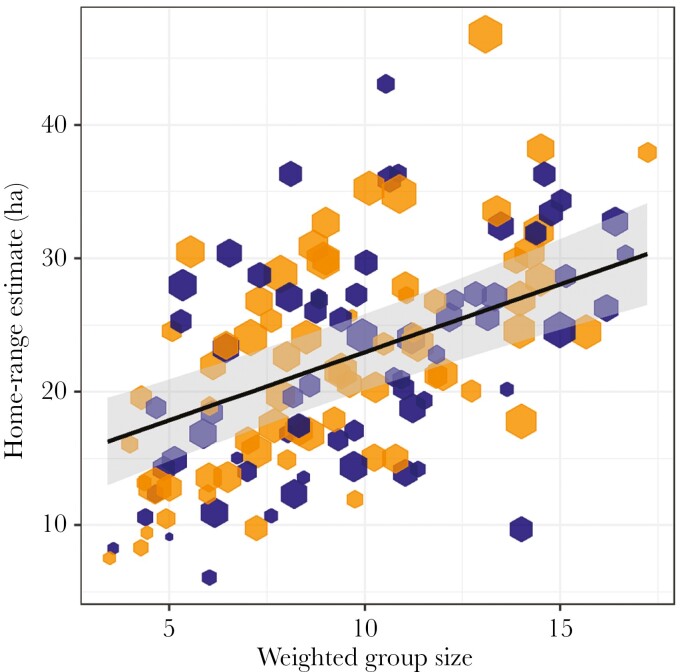
Relationship between group size and home-range size across all group-seasons. Each hexagon represents one group-season (orange represents breeding seasons, blue represents non-breeding seasons) and its size is proportional to the number of days sampled within a season (breeding seasons: 21 to 88 d; non-breeding seasons: 20 to 74 d). Line and shaded area represent estimated marginal means and 95% confidence interval with respect to NDVI and number of days sampled. *N*_GroupSeasons_ = 133, *N*_Groups_ = 12, *N*_Seasons_ = 19.

Dwarf mongoose home ranges have a mean ± SE size (95% KDE) of 22.4 ± 0.7 ha (range = 6.1 to 46.8 ha). After controlling for a positive effect of number of days sampled (LRT: χ^2^_1_ = 8.10, *P* = 0.004), we found a significant effect of the interaction between group size and NDVI (LRT: χ^2^_1_ = 6.15, *P* = 0.01), such that larger groups occupied larger home ranges, but the slope of this relationship increased with increasing greenness. Averaging over NDVI values, each additional group member was related to a 1.0-ha (95% CIs: 0.7 to 1.3 ha) increase in home-range size (Fig. 2). There were no significant effects of season type or its interactions with group size or NDVI, nor a quadratic effect of group size, on home-range size (all χ^2^_1_ < 1.53, all *P* > 0.21; Table S1). These results were qualitatively the same with regards to group size, season type, and NDVI when modeled with AKDE derived home-range sizes (Table S2).

### Daily movements

**Fig. 3.**
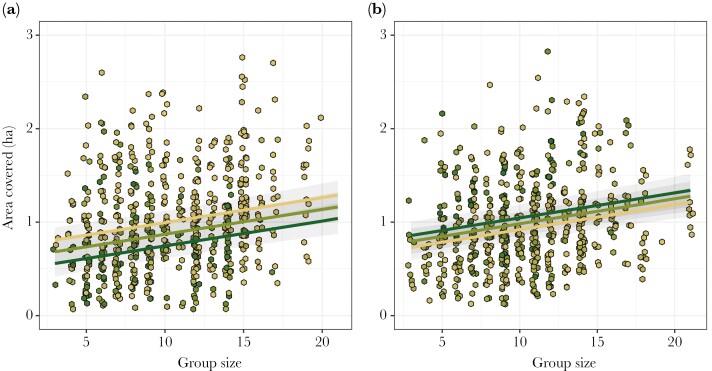
Relationship between area covered (ha) and group size, NDVI and season type: a) breeding season, b) non-breeding season. Lines and shading represent model predicted means and 95% confidence intervals for NDVI values of 0.3 (light brown), 0.4 (light green), and 0.5 (dark green). Points shaded by NDVI value. *N*_GroupDays_ = 1,192, *N*_Groups_ = 12, *N*_Seasons_ = 20.

Mongoose groups moved a total daily distance (as measured by total track length) of 1,536 ± 18 m (mean ± SE; range = 162 to 4,808 m), covering 1.0 ± 0.01 ha in area (range = 0.1 to 4.3 ha). Both total track length and area used were influenced by all 3 main predictors in the same way (Table S3). Larger groups had longer track lengths (LRT: χ^2^_1_ = 28.48, *P* < 0.001; Table S3a) and used larger daily areas (χ^2^_1_ = 40.29, *P* < 0.001; Table S3b; Fig. 3), such that each additional group member was related to a 26-m (95% CIs: 17 to 36 m) increase in track length and a 0.03-ha (0.02 to 0.04 ha) increase in area used. This group size relationship was best modeled as linear as there was no significant quadratic effect of group size on either track metric (track length: χ^2^_1_ = 2.94, *P* = 0.09; area used: χ^2^_1_ = 0.47, *P* = 0.49). There was, however, a significant effect of the interaction between NDVI and season type on both responses (track length: χ^2^_1_ = 29.18, *P* < 0.001; area used: χ^2^_1_ = 52.17, *P* < 0.001). For every 0.1 decrease in NDVI in the breeding season, groups moved 140 m (95% CIs: 108 to 172 m) further and used 0.13 ha (0.10 to 0.15 ha) more area (Fig. 3a); the same change in NDVI in the non-breeding season resulted in groups moving 24 m (−27 to 74 m) less far and using 0.18 ha (−0.05 to 0.32 ha) less area (Fig. 3b), although both of these effects in the non-breeding season have confidence intervals spanning zero (indicating no clear effect).

**Fig. 4.**
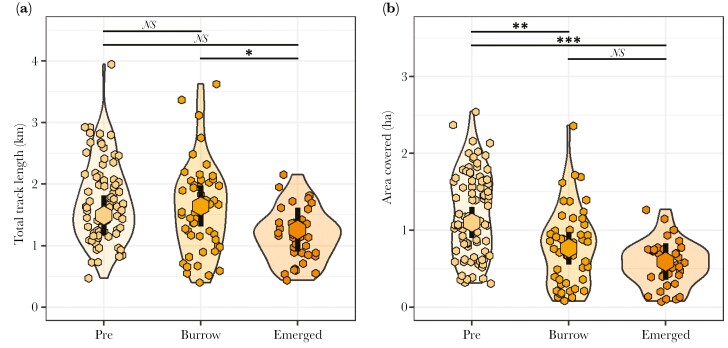
Track metrics split between the 3 breeding-season periods. *Pre*: the 28 d before the litter was born; *Burrow*: the 28 d following their birth when the pups are constrained to stay at the burrow; *Emerged*: the following 28 d (29 to 56 d post-birth) when the pups begin to travel with the group. Central points and lines represent estimated marginal means and 95% CIs for each period conditioned on NDVI and group size. ***P < 0.001, **P < 0.01, *P < 0.05, *NS* = nonsignificant at α = 0.05. *N*_GroupDays_ = 170, *N*_Groups_ = 12, *N*_Seasons_ = 9.

There were significant relationships between specific 4-wk periods within the breeding season and movement characteristics, controlling for group size and NDVI (Table S4). Total track length was affected by breeding-season period (LRT: χ^2^_2_ = 8.69, *P* = 0.01; Table S4a; Fig. 4a): posthoc testing (Table S5a) revealed that track length was shorter when groups were moving with recently emerged pups (*Emerged* period) than when the pups were dependent at the burrow (*Burrow* period; Tukey: *t*_162_* *= 2.88, *P* = 0.01), although there was no significant difference in this metric between *Pre* and *Burrow* periods (*t*_162_* *= −1.14, *P* = 0.49), nor the *Burrow* and *Emerged* periods (*t*_161_* *= 1.47, *P* = 0.31). There was also strong evidence that area covered was affected by breeding-season period (LRT: χ^2^_2_ = 19.82, *P* < 0.001; Table S4b; Fig. 4b): posthoc testing (Table S5b) revealed that mongoose groups covered a larger area in the *Pre* period than in either the *Burrow* (Tukey: *t*_158_ = 3.31, *P* = 0.003) or *Emerged* (*t*_132_ = 4.22, *P* < 0.001) periods, but there was no significant difference in area covered between the *Burrow* and *Emerged* periods (*t*_160_ = 1.71, *P* = 0.21).

### Burrow use

**Fig. 5.**
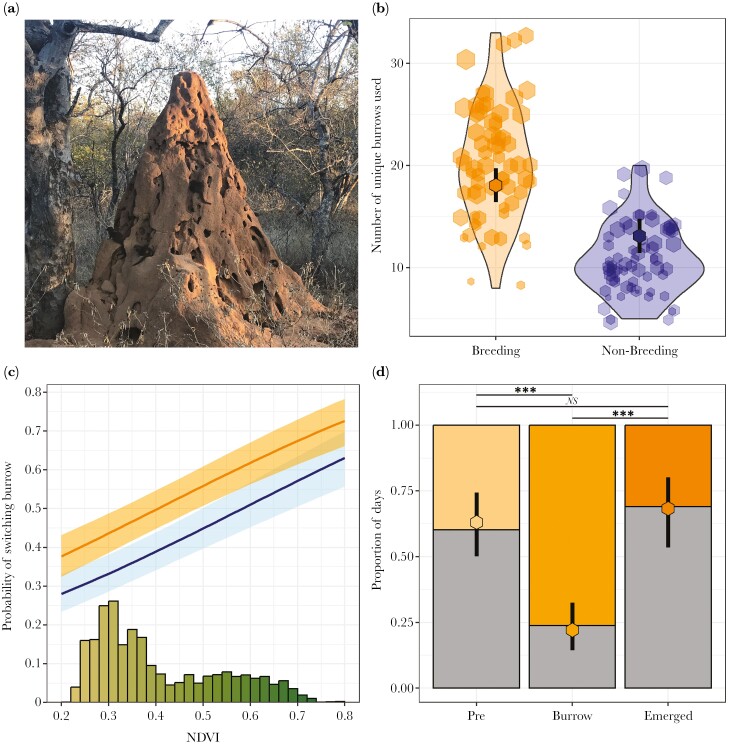
a) A termite mound sleeping burrow; termite mounds accounted for 73% of all sleeping burrows used (3,108/4,274 observed). b) Number of burrows used by a group per season. Hexagon size is proportional to the number of observation days in the specific group-season, ranging from 21 to 72 in the non-breeding season, and from 21 to 95 in the breeding season. Central point represents estimated marginal mean for each season conditioned over NDVI, group size and days sampled, arms represent 95% CIs. *N*_GroupSeasons_ = 133, *N*_Groups_ = 12, *N*_Seasons_ = 19. c) The proportion of days that a group changed burrows per season type. Histogram denotes the distribution of NDVI data. *N*_GroupDays_ = 2,914, *N*_Groups_ = 12, *N*_Seasons_ = 18. b–c) Orange represents breeding season, blue represents non-breeding season. d) Likelihood of switching burrow for the 3 breeding-season periods. Gray bars represent staying at the same burrow, colored bars represent switching burrow. Points and arms represent estimated marginal means conditioned on NDVI and group size, arms represent 95% CIs. ***P < 0.001, *P < 0.05. *N*_GroupDays_ = 523, *N*_Groups_ = 12, *N*_Seasons_ = 8.

Mongoose groups used a mean ± SE of 15.5 ± 0.6 burrows (such as termite mounds: Fig. 5a) per season (range = 5 to 33). After controlling for a positive effect of the number of days sampled (LRT: χ^2^_1_ = 54.30, *P* < 0.001), the main effect of season type was the only significant predictor of number of burrows used in a given season (χ^2^_1_ = 14.12, *P* < 0.001; Table S6a): groups used a mean of 5 more burrows (95% CIs: 2.7 to 7.2) in a breeding than a non-breeding season (Fig. 5b). There were no significant effects of group size or its interaction with season type, nor of NDVI or its interaction with season type, nor the interaction between group size and NDVI (all χ^2^_1_ < 0.98, all *P* > 0.22; Table S6a).

Mongoose groups regularly switched burrow, sleeping in a different burrow to the one they awoke in on 46 ± 20% (mean ± SE) of days. The likelihood of switching burrow was influenced by both season type (LRT: χ^2^_1_ = 8.99, *P* = 0.003; Table S6b) and NDVI (χ^2^_1_ = 69.66, *P* < 0.001), but there was no significant interaction between these factors (χ^2^_1_ = 3.21, *P* = 0.07). Groups were 1.6× more likely (95% CIs: 1.2 to 2.1×) to switch in the breeding season compared to the non-breeding season, while each 0.1 increase in NDVI increased likelihood of switching by 1.28× (1.21 to 1.36×) (Fig. 5c). There was no significant main effect of group size on likelihood of switching burrow, nor significant interactions between either group size and season type or between group size and NDVI (all χ^2^_1_ < 2.33, all *P* > 0.12; Table S6b).

Breeding-season period was also related to probability of switching burrow (LRT: χ^2^_2_ = 88.13, *P* < 0.001; Table S7; Fig. 5d), controlling for NDVI and group size. Posthoc testing (Table S8) revealed that mongooses were less likely to switch when they had dependent offspring at the burrow (*Burrow* period) than either before breeding (*Pre* period; Tukey: *t* = 6.88, *P* < 0.001) or once the pups had emerged and were moving with the group (*Emerged* period: *t* = −6.95, *P* < 0.001). There was, however, no significant difference in the likelihood of switching between the *Pre* and *Emerged* periods (*t* = −0.69, *P* = 0.77).

## Discussion

Using long-term data from a wild population, we demonstrate how group size, breeding seasonality, and environmental greenness have pronounced effects on the use of key resources and associated movements of dwarf mongooses. When considering home-range size and daily movements, group size was a consistently important predictor, while greenness and breeding seasonality had more nuanced effects. However, when considering the use of burrows, a non-depletable type of resource, breeding season and greenness had strong effects, with no detectable effect of group size. We also saw strong impacts of the presence of dependent offspring on space and burrow use, highlighting potentially hidden costs of breeding.

Bigger groups had larger home ranges and moved further per day than smaller groups, in line with our predictions based on the ecological constraints model (McNab 1963; Chapman and Chapman 2000a). The positive relationship between group size and daily movements highlights that larger mongoose groups not only traveled larger distances but also covered a larger unique area. This reflects the increased patch depletion resulting from the presence of more foraging individuals. One consequence of needing to move further is that larger groups likely expend more energy. However, this cost will be at least partially buffered by the anti-predator benefits of larger groups (Lima 1995). In the case of dwarf mongooses, this includes a larger proportion of time with a sentinel present—individuals within the foraging group regularly perform sentinel behavior, becoming vigilant from a raised position to scan for potential predators (Kern and Radford 2013, 2014)—with each individual able to contribute less when there are more potential cooperators (Ridley and Raihani 2007; Bednekoff 2015; Arbon et al. 2020).

Contrary to our predictions, there were no strong differences between the breeding and non-breeding seasons in home-range size, nor a clear effect of greenness on home-range size. Although environmental greenness appears to enable larger groups to hold larger areas when conditions were plentiful, this effect was not uniform across group sizes. There are a few potential explanations for such a lack of strong effects. First, as proposed in meerkats (Bateman et al. 2015), dwarf mongooses may defend a minimum required space for when food resources are scarce, even when seasonal changes make those resources more abundant. It is also possible that mongooses are defending a space containing a different limiting resource and food abundance is always adequate, as suggested for green woodhoopoes (*Phoeniculus purpureus*) with respect to roost holes (Radford and Du Plessis 2003). Burrows have been suggested as a limiting resource in other parts of the dwarf mongoose’s geographical range (Rasa 1986b), but the extremely high termite mound abundance at our study site renders this an unlikely explanation. Further research into the difference in changes to home-range size between territorial and nonterritorial species under different resource regimes may therefore reveal different relationships between area usage and resource abundance due to the costs of maintaining exclusive access to space.

Both movement characteristics were strongly linked to environmental greenness but only in the breeding season. During this season, groups moved further when the environment was less green, and therefore likely to be less productive. This is in accordance with our predictions and previous work on other species (Duncan et al. 2015; Seigle-Ferrand et al. 2021), demonstrating that as resource abundance increases, groups need to move less far to satisfy their energetic needs. The lack of this relationship in the non-breeding season could be due to the combination of breeding costs and a lag between primary and secondary productivity. The greener months of the non-breeding season immediately follow breeding, so groups may be foraging more intensely to recover body condition and prepare for the more arid non-breeding season. Similarly, the browner months of non-breeding come straight after greener months, so any lag between primary productivity (greenness) and secondary productivity (prey) could further mask the predicted relationship. Distance moved will also be influenced by factors that remain unaffected by breeding, such as the need to travel to latrine sites to mark territorial boundaries, the location of which persist across seasons and environmental conditions.

Our findings show that the best-fit of the relationships between dwarf mongoose group size and both home-range size and daily movement is linear. This contrasts the quadratic relationship, and thus an intermediate optimal group size, found in baboons and vulturine guineafowl (Markham et al. 2015; Papageorgiou and Farine 2020). Differences in ecology between the species provide candidate explanations. It was suggested that big groups of vulturine guineafowl may suffer from the costs of collective action, meaning that they occupied smaller home ranges similar in size to those of small groups (Papageorgiou and Farine 2020). However, in dwarf mongoose groups, the dominant pair exert disproportionate influence on group decisions (Cobb et al. 2022), likely dampening collective action costs. For baboons, it was suggested that small groups may range more widely than intermediate ones due to the combined forces of predator and competitor avoidance (Markham et al. 2015). In the case of dwarf mongooses, predation pressure is unlikely to be a key driver of increased movements at the home-range scale. The home ranges of martial eagles, a commonly seen predator, are over 100 km^2^ (van Eaden et al. 2017). The distances required to have a meaningful change in the predation pressure past the immediate term (e.g. fleeing from an observed threat) is therefore likely to be much too large.

When splitting the breeding season into functional periods, it becomes apparent that groups appear to be more constrained in their movements once they have dependent offspring, as seen in other group-living species (Papageorgiou and Farine 2020). When dependent offspring remain at the burrow, foraging of the group exhibited more central-place characteristics (Bell 1990); total track length was not altered, but area covered reduced as groups are constrained to return to the burrow, potentially multiple times a day, to exchange babysitters (Rood 1978; Rasa 1987). Once the pups have emerged, the group is constrained in a different way to move shorter distances than when pups were at the burrow whilst continuing to cover less area than before pups were born, likely due to the slower movement speeds and naivety of pups. This highlights a rarely considered potential cost of reproduction: more limited movement will increase patch depletion and therefore potentially decrease individual foraging success per unit time. This is a cost that impacts not just the breeding pair but all group members, as mongoose groups need to remain cohesive in a harsh environment (Cobb et al. 2022). In our population, these constraints may be less pronounced for later litters (we only considered the first litter of each season), as reproductive costs may be offset by increasing greenness throughout the breeding season. However, the additive costs of multiple breeding efforts may cancel out or even compound movement costs of breeding. More broadly, movement costs of offspring are likely to be widespread in the animal kingdom, such as in ungulates whose young follow their mother (Atmeh 2023) or birds that migrate with naïve offspring (Byholm et al. 2022).

Season had a pronounced effect on burrow usage, with mongoose groups using more unique burrows in the breeding season, and switching burrows more frequently, than in the non-breeding season. Groups also switched more frequently with increasing greenness, but this was independent of breeding season. These findings are generally in line with our predictions that groups would need to move more frequently when parasite and predator pressures are increased (Roper et al. 2002; Hockey et al. 2005; Lutermann et al. 2015; Strandburg-Peshkin et al. 2019). Increased parasite loads have been demonstrated to reduce reproductive success in African ground squirrels (Hillegass et al. 2010), so mongooses may be even more incentivized to avoid parasite pressure in the breeding season, although we did not observe the predicted increase in responsiveness to greenness when breeding. Contrary to our predictions, the detected increase in burrow switching during breeding was lessened when groups had small, immobile pups. In contrast to movement patterns, where the largest difference was between pre-birth and later time periods (i.e. *Burrow* and *Emerged*), burrow switching was greatly suppressed by the presence of pups that had not yet emerged. This is likely due to the costs of moving dependent offspring; mongoose pups are carried between burrows by adults in a coordinated manner that requires the cooperation of the entire group. The act of transporting pups is both energetically costly and vocally conspicuous (as groupmates need to communicate with one another) and therefore likely renders both adults and pups vulnerable to predation. We therefore suggest that these costs of moving pups must outweigh any potential detection and parasitism costs of remaining in the same location for extended periods. Contrary to our predictions, there was also no effect of group size on the number of burrows nor on likelihood on switching. This could suggest that any negative detection effects of larger groups are balanced by their greater ability to detect predators, but further work is required to test these relationships.

In summary, dwarf mongoose resource use and movements are significantly impacted by group size, breeding seasonality and environmental productivity. Group size has a larger impact on group movements and subsequent home ranges, likely due to the depletion of foraging resources, lending support to the core principle that increased group sizes result in increased competition and need for larger spaces. This contrasts with the relationships seen when considering nondepletable burrows where groups of all sizes behave similarly, and breeding seasonality becomes more important. Environmental conditions added nuance to our understanding of use of both resource types, with environmental greenness impacting burrow switching year-round and movements during breeding. Targeting specific behavioral windows around breeding enabled us to further our understanding of these relationships. Future work that moves past broadscale relationships, instead focusing on how groups move within their boundaries and relative to one another, will take us a step further into explaining how animal groups find, use, and defend resources vital to their survival and reproduction. Together, our results highlight how animals need to navigate the constraints of group-living and varying environmental conditions when making decisions about resources, decisions which are complicated by the need to produce and care for offspring.

## Supplementary Material

arae082_suppl_Supplementary_Material

## Data Availability

Analyses reported in this article can be reproduced using the data provided by Arbon et al. (2024).
