# Supplementary material for: Social and seasonal variation in dwarf mongoose home-range size, daily movements, and burrow use
Source: Behav Ecol. 2024 Oct 5;35(6):arae082. doi: 10.1093/beheco/arae082 (PMC11520750; doi:10.1093/beheco/arae082)
Supplement: arae082_suppl_Supplementary_Material [file arae082_suppl_supplementary_material.docx]

Social and seasonal variation in dwarf mongoose home-range size, daily movements and burrow use

Josh J Arbon, Amy Morris-Drake, Julie M Kern, Luca Giuggioli, Andrew N Radford

Supplementary Material

*Variation
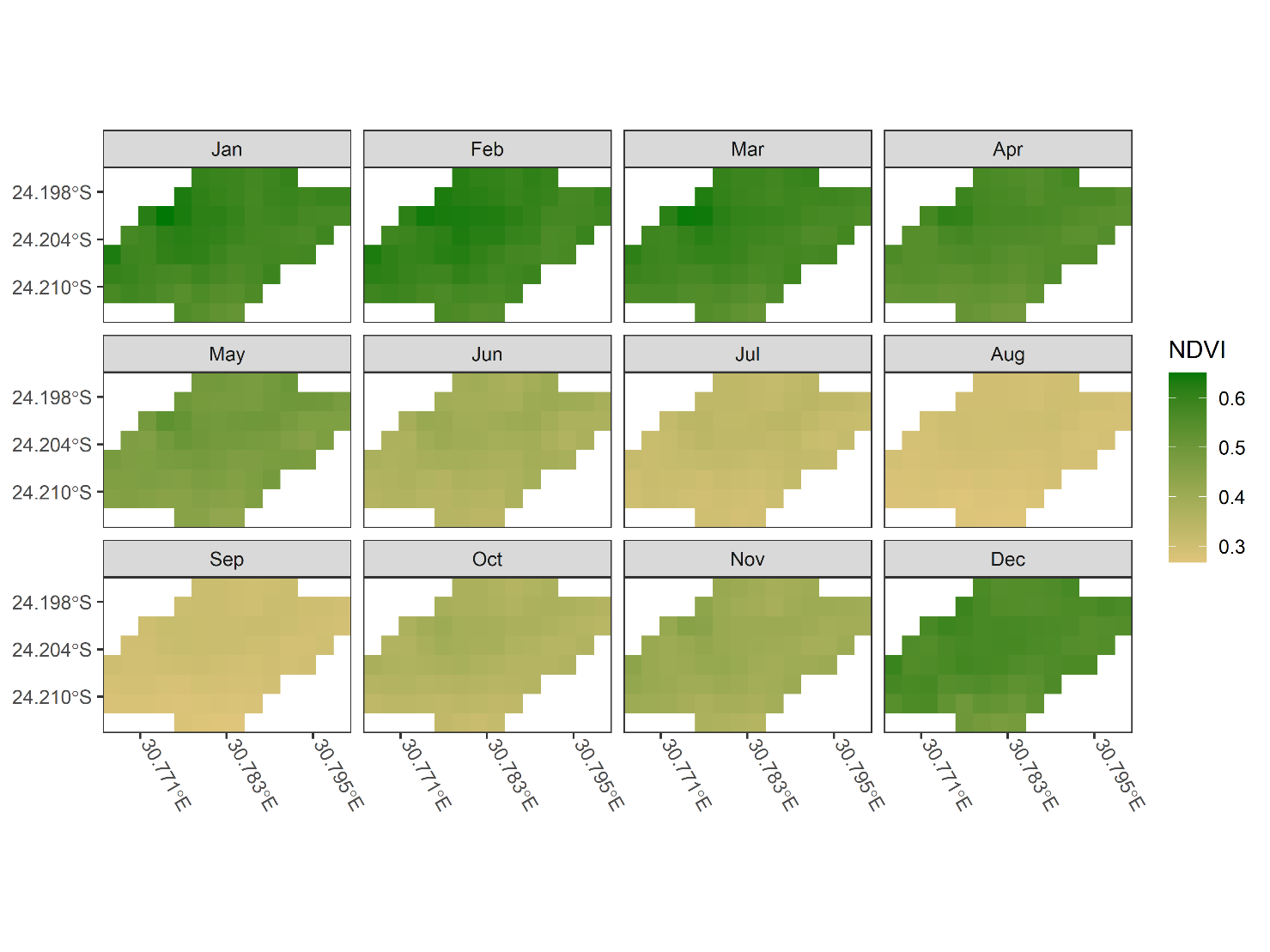
 in NDVI*

Supplementary Figure S1. Normalized difference vegetation index (NDVI) across the study site. Each pixel is 250 m x 250 m and the NDVI value represents the mean for that month across the study period 2013–2023.

*Kernel Density Estimation*

In addition to home-ranges calculated using kernel density estimation (KDE) presented in the main text, home-ranges were separately calculated using the autocorrelated kernel density estimation (AKDE) method by fitting continuous time movement models (ctmms) in the ‘ctmm’ R package (Calabrese et al. 2016), with the weighted, anisotropic Ornstein-Uhlenbeck foraging (OUF) model used. There was a strong correlation of 0.91 between home-range sizes calculated using the KDE and AKDE methods (Supplementary Figure S2), but the AKDE method estimated home-ranges to be 1.54x (SE = 0.02) larger than the original KDE method employed, incorporating space we have never observed being used by our study groups. This is perhaps because the OUF model assumes that the central tendency increases with distance from the centroid. For species such as dwarf mongooses that regularly scent-mark the periphery of their home-range to signal their presence to neighbours (Christensen et al. 2016), this assumption is likely unfounded. We therefore present output from original KDE models in the main text; outputs from ADKE models were qualitatively identical and presented in Supplementary Table S2.


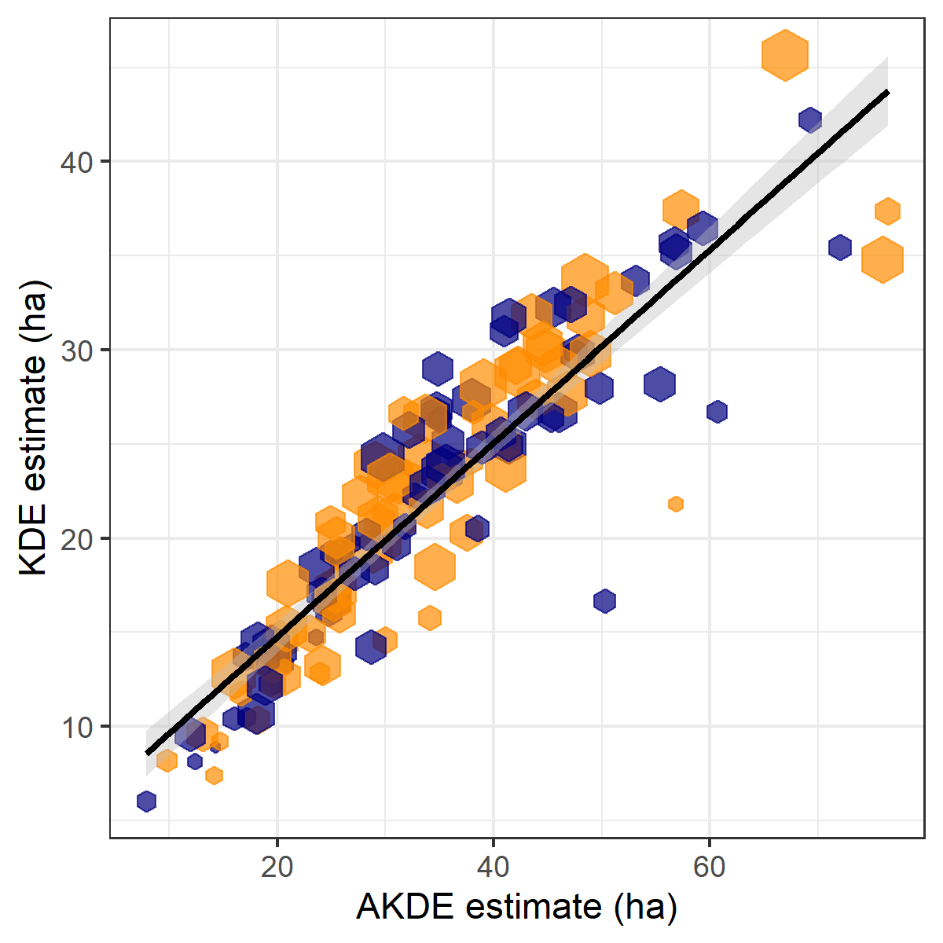


Supplementary Figure S2. Comparison of 95% homerange estimates over group-seasons calculated using independent identically distributed kernel density estimates (KDE) and autocorrelation kernel density estimation (ADKE) via fitting of continuous time movement modelling approaches. Orange points are breeding seasons, blue points are non-breeding seasons. Point size proportional to the number of sampling days in a season. KDE and AKDE estimates are tightly correlated, although AKDE estimates are larger in absolute value by a mean of 1.54x. N_GroupSeasons_ = 133, N_Groups_ = 12, N_Seasons_ = 19. Pearson’s product-moment correlation: r = 0.91, t_131_ = 24.93, P < 0.001.

*Main text model output tables*

In all tables, terms with estimates and confidence intervals (CIs) represent those present in the final model, significant values given in bold. Estimates and CIs for wGroup Size, Group Size and NDVI represent the estimates of the effects of scaled, centered variables such that mean = 0 and sd = 1.

**Supplementary Table S1.** Linear mixed model output with 95% kernel density estimate (in hectares) as home-range size predictor. N_GroupSeasons_ = 133, n_Groups_ = 12, n_Seasons_ = 19. ‘wGroup Size’ is weighted group size; ‘NB’ is non-breeding season, reference level for season type is ‘Breeding’. Random terms italicised; *χ^2^*, degrees of freedom and p values derived from likelihood ratio tests.

| Factor | Estimate | 95% CIs | *χ^2^* | df | P |
| --- | --- | --- | --- | --- | --- |
| Intercept | 15.47 | 9.71 – 21.33 |  |  |  |
| wGroup Size | 3.43 | 2.45 – 4.50 |  |  |  |
| wGroup Size^2^ |  |  | 1.31 | 1 | 0.25 |
| NDVI | 1.59 | -0.53 – 3.69 |  |  |  |
| Season Type _(NB)_ | 2.54 | -1.86 – 6.97 | 1.35 | 1 | 0.25 |
| wGroup Size : NDVI | 1.09 | 0.23 – 1.91 | 6.15 | 1 | **0.01** |
| wGroup Size : Season Type _(NB)_ |  |  | 0.01 | 1 | 0.91 |
| Season Type _(NB)_ : NDVI |  |  | 1.52 | 1 | 0.22 |
| Days Sampled | 0.13 | 0.04 – 0.21 | 8.10 | 1 | **0.004** |
| *Season* | *3.49* | *1.93 – 6.49* |  |  |  |
| *Group* | *3.72* | *3.97 – 5.25* |  |  |  |

**Supplementary Table S2.** Linear mixed model output with 95% autocorrelated kernel density estimate (in hectares) as home-range size predictor. N_GroupSeasons_ = 133, n_Groups_ = 12, n_Seasons_ = 19. ‘wGroup Size’ is weighted group size; ‘NB’ is non-breeding season, reference level for season type is ‘Breeding’. Random terms italicised; *χ^2^*, degrees of freedom and p values derived from likelihood ratio tests.

| Factor | Estimate | 95% CIs | *χ^2^* | df | P |
| --- | --- | --- | --- | --- | --- |
| Intercept | 29.63 | 18.96 – 40.36 |  |  |  |
| wGroup Size | 4.51 | 2.53 – 6.72 |  |  |  |
| NDVI | 2.77 | -0.88 – 6.39 |  |  |  |
| Season Type _(NB)_ | 2.63 | -4.98 – 10.30 | 0.50 | 1 | 0.48 |
| wGroup Size : NDVI | 2.1 | 0.37 – 3.80 | 5.62 | 1 | **0.02** |
| wGroup Size : Season Type _(NB)_ |  |  | 0.01 | 1 | 0.92 |
| Season Type _(NB)_ : NDVI |  |  | 3.34 | 1 | 0.07 |
| Days Sampled | 0.06 | -0.10 – 0.22 | 0.53 | 1 | 0.47 |
| *Season* | *5.56* | *2.29 – 8.09* |  |  |  |
| *Group* | *7.03* | *3.72 – 12.13* |  |  |  |

**Supplementary Table S3.** Linear mixed model outputs with a) total track length (m) and b) area covered (ha) as response terms. N_GroupDays_ = 1192, N_Gruops_ = 12, N_Seasons_ = 20. ‘NB’ is non-breeding season, reference level for season type is ‘Breeding’. Random terms italicised, *χ^2^*, degrees of freedom and p values derived from likelihood ratio tests.

| Factor | Estimate | 95% CIs | *χ^2^* | d.f. | P |
| --- | --- | --- | --- | --- | --- |
| a) Total track length (m) |  |  |  |  |  |
| Intercept | 1512.3 | 1354.00 – 1669.11 |  |  |  |
| Group Size | 99.63 | 63.50 – 136.01 | 28.48 | 1 | **<0.001** |
| Group Size^2^ |  |  | 2.94 | 1 | 0.09 |
| NDVI | -174.84 | -214.62 – -134.80 |  |  |  |
| Season Type _(NB)_ | -58.07 | -266.32 – 151.52 |  |  |  |
| Group Size : NDVI |  |  | 2.74 | 1 | 0.10 |
| Group Size : Season Type _(NB)_ |  |  | 1.26 | 1 | 0.26 |
| Season Type _(NB)_ : NDVI | 204.37 | 130.70 – 278.31 | 29.18 | 1 | **<0.001** |
| *Season* | *224.6* | *153.07 – 309.99* |  |  |  |
| *Group* | *87.28* | *44.37 – 158.56* |  |  |  |
| b) Area used (ha) |  |  |  |  |  |
| Intercept | 0.96 | 0.84 – 1.08 |  |  |  |
| Group Size | 0.10 | 0.07 – 0.13 | 40.29 | 1 | **<0.001** |
| Group Size^2^ |  |  | 0.47 | 1 | 0.49 |
| NDVI | -0.16 | -0.19 – -0.12 |  |  |  |
| Season Type _(NB)_ | -0.01 | -0.16 – 0.15 |  |  |  |
| Group Size : NDVI |  |  | 1.83 | 1 | 0.18 |
| Group Size : Season Type _(NB)_ |  |  | 0.09 | 1 | 0.77 |
| Season Type _(NB)_ : NDVI | 0.23 | 0.17 – 0.30 | 52.17 | 1 | **<0.001** |
| *Season* | *0.16* | *0.11 – 0.23* |  |  |  |
| *Group* | *0.09* | *0.05 – 0.16* |  |  |  |

**Supplementary Table S4.** Linear mixed model outputs for the track metrics split within breeding-season periods, with a) total track length (m) and b) area covered (ha) as response terms. N_GroupDays_ = 170, N_Gruops_ = 12, N_Seasons_ = 9. Random terms italicised, *χ^2^*, degrees of freedom and p values derived from likelihood ratio tests. Random effects fitted with weak Wishart prior to aid fitting. Pairwise comparison of breeding-season period levels given in Supplementary Table S5.

| Factor | Estimate | 95% CIs | *χ^2^* | d.f. | P |
| --- | --- | --- | --- | --- | --- |
| a) Total track length (m) |  |  |  |  |  |
| Intercept | 1523.57 | 1268.61 – 1786.47 |  |  |  |
| Group Size | 156.27 | 54.31 – 283.57 | 7.55 | 1 | **0.006** |
| NDVI | -92.27 | -216.94 – 40.23 | 1.84 | 1 | 0.18 |
| Breeding Season Period |  |  | 8.69 | 2 | **0.01** |
| Breeding Season Period _(Burrow)_ | 149.74 | -113.59 – 396.61 |  |  |  |
| Breeding Season Period _(Emerged)_ | -233.91 | -549.57 – 58.12 |  |  |  |
| *Season* | *341.30* | *131.42 – 526.78* |  |  |  |
| *Group* | *126.35* | *0.00 – Inf* |  |  |  |
| b) Area used (ha) |  |  |  |  |  |
| Intercept | 1.13 | 0.97 – 1.29 |  |  |  |
| Group Size | 0.13 | 0.02 – 0.22 | 8.35 | 1 | **0.003** |
| NDVI | -0.05 | -0.16 – 0.03 | 2.23 | 1 | 0.14 |
| Breeding Period |  |  | 19.82 | 2 | **<0.001** |
| Breeding Period _(Burrow)_ | -0.33 | -0.51 – -0.13 |  |  |  |
| Breeding Period _(Emerged)_ | -0.51 | -0.72 – -0.28 |  |  |  |
| *Season* | *0.14* | *0.04 – 0.31* |  |  |  |
| *Group* | *0.17* | *0.00 – 0.22* |  |  |  |

**Supplementary Table S5.** Post-hoc Tukey outputs for the track metrics across three breeding-season periods. Degrees of freedom were calculated using the Kenward-Roger method. Post-hoc tests applied to final models derived from Supplementary Table S4.

| Factor | Estimate | 95% CIs | *t* | d.f. | P |
| --- | --- | --- | --- | --- | --- |
| a) Total track length (m) |  |  |  |  |  |
| Pre \| Burrow | -150 | -803 – 504 | -1.14 | 162 | 0.49 |
| Pre \| Emerged | 234 | -447 – 915 | 1.47 | 161 | 0.31 |
| Burrow \| Emerged | 384 | -304 – 1071 | 2.88 | 162 | **0.01** |
| b) Area used (ha) |  |  |  |  |  |
| Pre \| Burrow | 0.33 | -0.08 – 0.75 | 3.31 | 158 | **0.003** |
| Pre \| Emerged | 0.51 | 0.07 – 0.95 | 4.22 | 132 | **<0.001** |
| Burrow \| Emerged | 0.17 | -0.28 – 0.63 | 1.71 | 157 | 0.21 |

**Supplementary Table S6.** Model outputs for burrow metrics: a) linear mixed model with number of unique burrows used by a group per season as the response (N_GroupSeasons_ = 133, n_Groups_ = 12, n_Seasons_ = 19); and b) generalised linear mixed model with a binary response for whether a group switched burrow on a particular day, fitted as a binomial model with a logit link function (N_GroupDays_ = 2914, N_Groups_ = 12, N_Seasons_ = 18). ‘wGroup Size’ is weighted group size; ‘NB’ is non-breeding season, reference level for season type is ‘Breeding’. Random terms italicised, *χ^2^*, degrees of freedom and p values derived from likelihood ratio tests.

| Factor | Estimate | 95% CIs | *χ^2^* | d.f. | P |
| --- | --- | --- | --- | --- | --- |
| a) Number of burrows |  |  |  |  |  |
| Intercept | 8.13 | 5.01 – 11.42 |  |  |  |
| wGroup Size | -0.1 | -0.73 – 0.56 | 0.07 | 1 | 0.79 |
| NDVI | 0.46 | -0.59 – 1.52 | 0.81 | 1 | 0.37 |
| Season Type _(NB)_ | -4.96 | -7.23 – -2.69 | 14.12 | 1 | **<0.001** |
| wGroup Size : NDVI |  |  | 0.52 | 1 | 0.47 |
| wGroup Size : Season Type _(NB)_ |  |  | 0.40 | 1 | 0.52 |
| Season Type _(NB)_ : NDVI |  |  | 0.97 | 1 | 0.23 |
| Days Sampled | 0.2 | 0.15 – 0.24 | 54.30 | 1 | **<0.001** |
| *Season* | *1.48* | *1.14 – 2.22* |  |  |  |
| *Group* | *0.7* | *0.00 – 1.66* |  |  |  |
| b) Burrow switching |  |  |  |  |  |
| Intercept | 0.01 | -0.20 – 0.22 |  |  |  |
| Group Size | -0.05 | -0.14 – 0.04 | 1.29 | 1 | 0.26 |
| NDVI | 0.33 | 0.25 – 0.41 | 69.66 | 1 | **<0.001** |
| Season Type _(NB)_ | -0.44 | -0.75 – -0.17 | 8.99 | 1 | **0.003** |
| Group Size : NDVI |  |  | 0.02 | 1 | 0.89 |
| Group Size : Season Type _(NB)_ |  |  | 2.32 | 1 | 0.13 |
| Season Type _(NB)_ : NDVI |  |  | 3.21 | 1 | 0.07 |
| *Season* | *0.22* | *0.12 – 0.38* |  |  |  |
| *Group* | *0.09* | *0.00 – 0.26* |  |  |  |

**Supplementary Table S7.** Generalised linear mixed model output for likelihood of switching burrow on a particular day (fitted as a binomial model with a logit link function) split within breeding-season periods. N_GroupDays_ = 523, N_Groups_ = 12, N_Seasons_ = 8. Random terms italicised, *χ^2^*, degrees of freedom and p values derived from likelihood ratio tests. Random effects fitted with weak Wishart prior to aid fitting. Pairwise comparison of breeding-season period levels given in Supplementary Table S8.

| Factor | Estimate | 95% CIs | *χ^2^* | d.f. | P |
| --- | --- | --- | --- | --- | --- |
| Intercept | 0.57 | -0.05 – 1.12 |  |  |  |
| Group Size | 0.02 | -0.28 – 0.30 | 0.04 | 1 | 0.84 |
| NDVI | 0.18 | -0.16 – 0.54 | 1.11 | 1 | 0.29 |
| Breeding Period |  |  | 88.13 | 2 | **<0.001** |
| Breeding Period _(Burrow)_ | -1.80 | -23.9 – -1.29 |  |  |  |
| Breeding Period _(Emerged)_ | 0.23 | -0.52 – 0.93 |  |  |  |
| *Season* | *0.21* | *0.18 – 0.60* |  |  |  |
| *Group* | *0.60* | *0.29 – 1.07* |  |  |  |

**Supplementary Table S8.** Post-hoc Tukey outputs for the likelihood of switching burrows across three breeding-season periods. Post-hoc test applied to final model derived from Supplementary Table S7.

| Factor | Estimate | 95% CIs | *z* | d.f. | P |
| --- | --- | --- | --- | --- | --- |
| Pre \| Burrow | 1.80 | 0.74 – 2.85 | 6.88 | Inf | **<0.001** |
| Pre \| Emerged | -0.23 | -1.39 – 0.93 | -0.69 | Inf | 0.77 |
| Burrow \| Emerged | -2.03 | -3.19 – -0.87 | -6.95 | Inf | **<0.001** |

*References*

Calabrese JM, Fleming CH, Gurarie E. 2016. ctmm: an r package for analyzing animal relocation data as a continuous-time stochastic process. Methods in Ecology and Evolution. 7(9):1124–1132. doi:10.1111/2041-210X.12559.

Christensen C, Kern JM, Bennitt E, Radford AN. 2016. Rival group scent induces changes in dwarf mongoose immediate behavior and subsequent movement. Behavioral Ecology. 27(6):1627–1634. doi:10.1093/beheco/arw092.
